# Personality dimensions and coping strategies in remitted bipolar disorder

**DOI:** 10.1192/j.eurpsy.2021.1664

**Published:** 2021-08-13

**Authors:** E. Mhiri, N. Messedi, W. Bouattour, F. Charfeddine, J. Aloulou

**Affiliations:** Psychiatry (b), Hedi Chaker University hospital, sfax, Tunisia

**Keywords:** Personality, coping, bipolar, remitted

## Abstract

**Introduction:**

The influence of personality on how people deal with stressful situations has long been discussed. In bipolar disorder, these two entities seem to have a role in the outcome of the disease.

**Objectives:**

To study the relationships between coping strategies in stressful situations and personality dimensions in euthymic bipolar patients.

**Methods:**

This is a cross-sectional, descriptive and analytical study of 30 patients followed for bipolar disorder in remission, at the psychiatric outpatient clinic at the Hédi Chaker Uuniversity Hospital in Sfax. We used a socio-demographic and clinical data sheet and the Ten Items Personality Inventory (TIPI) to evaluate personality dimensions and the Ways Of Coping Checklist (WWC) for the assessment of coping.

**Results:**

The mean age of the patients was 43.77 years, the sex ratio was 0.5. Bipolar I disorder was diagnosed in 93% of patients. WCC : -Coping focused on the problem : 70% of the patients. -Emotion-centered coping : 20% of patients -Coping focused on seeking social support : 10% of patients. TIPI : Conciousness was the most represented trait of personality (36.7%), agreableness (30%) and extraversion (20%). Extraversion was associated with coping focused on the problem: (p=0.015). Agreableness was associated with coping focused on seeking social support:(p=0.033).

**Conclusions:**

Our study showed that conciousness is the most common trait of personality in bipolar disorder patients. The coping focused on the problem is the most frequent strategy which correlated with extraversion, so, personality dimensions appear as a target for cognitive interventions.

**Disclosure:**

No significant relationships.

